# Synergistic role of plant growth-promoting rhizobacteria and fungi in biofertilizer development for chilli (*Capsicum annuum* L.): mechanistic and functional insights

**DOI:** 10.3389/fpls.2026.1844723

**Published:** 2026-06-17

**Authors:** Pushpa Gehlot, Jyoti Yadav, Tripta Jain

**Affiliations:** Department of Botany, Mohanlal Sukhadia University, Udaipur, Rajasthan, India

**Keywords:** biofertilizers, *Capsicum annuum*, microbial consortia, nutrient cycling, PGPR-PGPF synergy, sustainable agriculture

## Abstract

Agricultural sustainability is increasingly threatened by the excessive use of chemical fertilizers, which has led to soil degradation, nutrient imbalance, and ecological disturbances, necessitating the development of eco-friendly alternatives. Biofertilizers based on plant growth-promoting microorganisms (PGPM), particularly plant growth-promoting rhizobacteria (PGPR) and plant growth-promoting fungi (PGPF), have emerged as promising tools for enhancing crop productivity while maintaining soil health. Chilli (*Capsicum annuum* L.), an economically important spice crop, is often constrained by nutrient deficiencies, declining soil fertility, and disease pressure, which significantly affect its yield and quality. This review critically examines current advances in the mechanistic and functional roles of PGPR and PGPF in sustainable chilli cultivation, with particular emphasis on microbial-mediated nutrient mobilization, including nitrogen fixation, phosphate solubilization, phytohormone production, enzymatic activities, and biocontrol mechanisms. Emerging evidence suggests that microbial consortia involving bacterial and fungal inoculants establish synergistic interactions that enhance nutrient availability, improve root architecture, and increase plant tolerance to biotic and abiotic stresses more effectively than single inoculants. Additionally, microbial traits such as siderophore production, and antagonistic potential further contribute to enhanced plant growth, nutrient-use efficiency, and yield stability. Particular attention is also given to recent developments in consortium-based biofertilizer formulations, including carrier selection, inoculant stability, microbial survival, and field-level applicability. Collectively, the available literature indicates that integrated PGPR-PGPF biofertilizer approaches represent a promising strategy for improving chilli productivity, reducing dependency on chemical fertilizers, and promoting long-term soil health and sustainable agricultural resilience.

## Graphical Abstract

**Figure:**
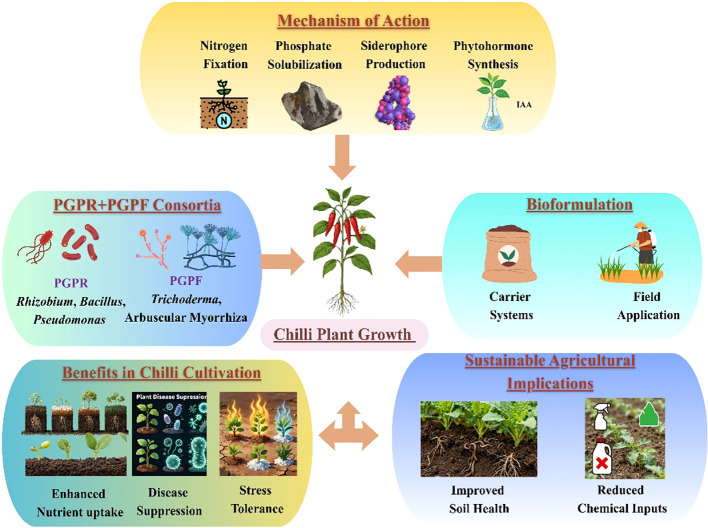

## 1 Introduction

Agriculture plays a pivotal role in global food security, yet it faces mounting challenges due to population growth, climate variability and the excessive reliance on chemical fertilizers. Although chemical inputs have substantially increased crop productivity, their indiscriminate application has led to severe environmental consequences such as soil acidification, loss of organic matter, nutrient leaching, water pollution and disruption of beneficial soil microbiota (Zafar et al., 2024; Islam, 2025). These impacts have raised global concerns about the long-term sustainability of conventional agricultural practices. Sustainable agriculture emphasizes the use of eco-friendly approaches that maintain soil fertility, conserve biodiversity and ensure productivity with minimal ecological harm (Gamage et al., 2023). In this regard, biofertilizers have gained significant attention as promising alternatives to chemical fertilizers. Biofertilizers are living microbial formulations that enhance plant growth by improving nutrient availability, facilitating nutrient uptake and stimulating plant physiological processes. Their application not only reduces the dependency on synthetic fertilizers but also improves soil health and contributes to environmental responsible farming (Ghimirey et al., 2024).

Plant growth-promoting microorganisms (PGPM), which include bacteria and fungi, have been thoroughly researched as biofertilizer agents because of their potential to increase crop output (Aloo et al., 2022). These microorganisms promote plant growth through diverse mechanisms, including nitrogen fixation, phosphate and potassium solubilization, production of siderophores, secretion of phytohormones such as indole-3-acetic acid (IAA), gibberellins and enhancement of plant tolerance to abiotic and biotic stresses (Kumar et al., 2022). Notable PGPM include bacterial genera such as *Azospirillum*, *Bacillus*, *Pseudomonas* and *Rhizobium*, as well as beneficial fungi such as arbuscular mycorrhizal fungi (AMF) and *Trichoderma* species. Their capacity to improve plant vigor and resilience underscores their importance in sustainable crop production (Soumare et al., 2021).

Chilli (*Capsicum annuum* L.) is one of the most important spice crops globally and holds immense economic and nutritional value. However, chilli cultivation is constrained by nutrient limitations, soil degradation and susceptibility to diseases, which often result in yield reduction (Kurubetta et al., 2017). The use of PGPM-based biofertilizers in chilli production offers an eco-friendly strategy to overcome these challenges by improving nutrient availability and enhancing plant defense mechanisms (Mohanty et al., 2021).

Review of literature suggests that PGPMs have been extensively investigated for their biocontrol potential. However, comparatively fewer studies have focused on their application as plant growth promoters and biofertilizers. Recent studies have demonstrated the promising role of microbial consortia involving PGPR and PGPF in enhancing plant growth, nutrient acquisition, and stress tolerance through synergistic interactions. Nevertheless, comprehensive investigations integrating PGPR and PGPF for the development of efficient consortium-based biofertilizers in economically important cash crops such as chilli (*C. annuum*) remain limited. Therefore, the present investigation focuses on the screening of PGPMs for plant growth-promoting attributes such as nitrogen fixation, phosphate solubilization, HCN production, siderophore production, and phytohormone production. In addition, the study aims to evaluate the synergistic plant growth-promoting potential of PGPR-PGPF consortium-based biofertilizers formulated using suitable organic and inorganic carriers under *in vivo* conditions.

## 2 Chilli: an overview

Chilli (*C. annuum*) is one of the most widely cultivated spice and vegetable crops, which belongs to the family Solanaceae. It is globally recognized for its pungency, flavor and high nutritional value. Chilli fruits are rich sources of bioactive compounds such as capsaicinoids, carotenoids, ascorbic acid and phenolic compounds, which contribute not only to their characteristic taste and color but also to their medicinal and nutraceutical properties (Kumar et al., 2022). These attributes make chilli a crop of dual importance, serving both culinary and therapeutic purposes. From an economic perspective, chilli holds a significant position in the global spice trade and agricultural economies. It is cultivated extensively across tropical and subtropical regions, with major producers including India, China, Mexico and Thailand. India is recognized as the largest producer, consumer and exporter of chilli, contributing substantially to its agricultural export earnings (Mohd Ali et al., 2025). The crop’s adaptability to diverse agro-climatic conditions has enabled its widespread cultivation, ranging from subsistence farming to intensive commercial production systems. As a spice crop, chilli is indispensable in culinary practices worldwide, forming the basis of numerous cuisines by enhancing flavor, color and pungency (Mi et al., 2020). In addition to its role as a spice, chilli is consumed as a fresh vegetable, processed into pickles, sauces and powders, or used in value-added food products. The global demand for processed chilli products continues to expand, reflecting changing dietary preferences and the growing popularity of spicy foods (Dutta and Liklam Loushigam, 2026).

Beyond its role in food and trade, chilli cultivation provides livelihood and income security for millions of small and marginal farmers. Its high market value, combined with short cropping duration and multiple harvests per season, makes chilli a profitable crop choice (Glory and Masih, 2025). Moreover, the rising demand in pharmaceutical and nutraceutical industries for capsaicin and related bioactive compounds further enhances the economic potential of chilli. Thus, chilli is not only a crop of culinary importance but also a driver of rural economies and global trade. Its economic significance, coupled with its nutritional and industrial value, underscores the need for sustainable cultivation practices to ensure consistent productivity and profitability (Esakkiammal et al., 2024.).

### 2.1 Nutritional and medicinal value

Chilli (*C. annuum*) is valued not only for its pungency and culinary applications but also for its remarkable nutritional and medicinal properties. The fruit contains a diverse array of bioactive compounds including vitamins, minerals, alkaloids and antioxidants, which contribute significantly to human health (Mohd Ali et al., 2025).

One of the most characteristic compounds of chilli is capsaicin, an alkaloid responsible for its pungency. Capsaicin and its analogs, collectively known as capsaicinoids, exhibit multiple pharmacological properties including analgesic, anti-inflammatory, anti-obesity, anti-diabetic and anticancer activities (Devrani et al., 2024). Capsaicin is widely used in pharmaceutical formulations such as topical creams, transdermal patches and pain-relief medications due to its ability to desensitize sensory neurons and alleviate chronic pain. Additionally, emerging evidence suggests its role in modulating metabolism and cardiovascular health (Maharjan et al., 2024).

Chilli fruits are also rich in essential vitamins, particularly vitamin C (ascorbic acid), which functions as a potent antioxidant and plays a critical role in enhancing immunity, collagen synthesis and wound healing. Vitamin A, derived from chilli carotenoids such as β-carotene, supports vision, growth and immune defense. Furthermore, chilli provides moderate amounts of vitamin E, vitamin B6 and folates, all of which contribute to metabolic and physiological functions (Mohd Ali et al., 2025). In terms of antioxidant potential, chilli contains carotenoids (capsanthin, capsorubin and β-carotene), flavonoids and phenolic compounds, which scavenge free radicals and reduce oxidative stress. These antioxidants are associated with reduced risks of chronic diseases including cardiovascular disorders, neurodegenerative conditions and certain cancers. The high antioxidant activity of chilli also contributes to its nutraceutical importance and value in functional foods (Zhou et al., 2025). Medicinally, chilli has been conventionally used in several cultures for its therapeutic benefits, including appetite stimulation, digestion enhancement and as a natural remedy for common colds and respiratory ailments. Modern pharmacological studies further validate these uses, emphasizing chilli’s potential in preventive healthcare and disease management (Aguilar-Meléndez et al., 2021).

Therefore, chilli represents a unique crop that integrates nutritional richness and therapeutic properties, making it not only an essential dietary component but also a valuable resource for the food, pharmaceutical and nutraceutical industries.

### 2.2 Global production trends and India’s contribution to chilli cultivation/

Chilli (*C. annuum*) is recognized as one of the most significant spice crops cultivated worldwide, owing to its multifaceted uses as a culinary ingredient, a natural colorant and a source of pharmacologically active compounds such as capsaicin. Its adaptability across diverse agro-climatic regions has made it a globally traded commodity and its demand is steadily increasing in both fresh and processed forms (Lakshmi et al., 2025). Chilli is widely cultivated across Asia, Africa, and other regions, reflecting its global agricultural and economic importance (Anantha and Sidana, 2019). However, inconsistencies exist in production data. Recent estimates report global chilli production at approximately 4.03 million tons, which is considerably lower than earlier claims exceeding 40 million tons. These discrepancies likely arise from differences in data reporting and the lack of clear separation between green and dried chilli production (Singh and Kumar, 2025). India is the world’s leading producer, consumer, and exporter, with an annual production of about 13.76 million tons (Geetha and K.Selvarani, 2017). Its contribution to global production ranges from 25–36%. Within India, chilli production is regionally concentrated, with Andhra Pradesh contributing 51-55% of total production despite a lower share of cultivated area, indicating higher productivity (Bansal et al., 2022a). Additionally, a few major states together account for up to 90% of national production.

Chilli is a key component of India’s spice export sector, contributing 38.43% of export volume and 31% of value (Bansal et al., 2022). Export quantities have increased significantly, exceeding 700,000 tons by 2024-25 (Pushparani et al., 2025). India holds about 25% of the global chilli market, generating nearly USD 1 billion annually. Major export destinations include China, the United States, Sri Lanka, Bangladesh, and the Gulf countries (Khan et al., 2025). Overall, while India’s dominance is well established, global production estimates require more standardized and updated reporting. However, chilli production is significantly constrained by recurrent biotic stresses, including anthracnose (fruit rot), leaf curl virus, bacterial leaf spot, powdery mildew, blight, and wilt, along with abiotic challenges arising from climate variability and declining soil fertility. Addressing these limitations through integrated pest management, improved cultivars and biofertilizer interventions could further enhance India’s contribution to the global chilli economy (Misal et al., 2022).

## 3 Constraints in chilli production

Although chilli (*C. annuum*) is a high-value crop with significant economic potential, its production is constrained by several interrelated agronomic and pathological challenges. Among these, nutrient deficiencies, declining soil health and persistent disease pressure are the most critical factors that limit productivity and quality (Chuichulcherm et al., 2013).

### 3.1 Nutrient deficiencies

Chilli is a nutrient-intensive crop, requiring balanced supplies of macronutrients (nitrogen, phosphorus, potassium) and micronutrients (zinc, boron, iron and magnesium) for optimum growth and fruit development. Deficiencies in these nutrients often lead to reduced plant vigor, poor fruit set and inferior pod quality (Shil et al., 2013). Nitrogen deficiency results in stunted growth and chlorosis, while inadequate phosphorus limits root development and flowering. Potassium deficiency is particularly detrimental, causing reduced fruit size, improper ripening and lower capsaicin content (Islam, 2025). Micronutrient imbalances such as zinc or boron deficiency, have been linked to flower drop and fruit deformation. The intensive use of chemical fertilizers without proper soil testing has further aggravated nutrient imbalances in chilli-growing regions (Yadav et al., 2023).

### 3.2 Soil health decline

Soil health in chilli production systems has steadily declined due to prolonged monocropping, overuse of chemical fertilizers, and insufficient incorporation of organic matter. The depletion of soil organic carbon, deterioration of soil structure and reduced microbial diversity have collectively lowered soil fertility and resilience (Țopa et al., 2025). Acidification, salinization and secondary nutrient depletion are increasingly reported from major chilli belts, particularly in South India. The reduced biological activity of soils not only affects nutrient cycling but also enhances the crop’s susceptibility to biotic stresses. This trend poses a significant challenge to the long-term sustainability of chilli cultivation (Khan et al., 2024).

### 3.3 Disease pressure

Chilli is highly vulnerable to a wide spectrum of pathogens, including viruses, fungi, bacteria and nematodes, which substantially reduce yields. Viral diseases such as chilli leaf curl virus (ChiLCV), mosaic virus and tobacco streak virus are widespread, often transmitted by vectors like whiteflies and thrips (Nalla et al., 2023). Among fungal pathogens, *Colletotrichum capsici* (causing anthracnose), *Alternaria alternata* (causing *Alternaria* Leaf Spot and Fruit Rot) and *Fusarium* species (causing Wilt) are particularly destructive. Bacterial leaf spot (*Xanthomonas campestris* pv. *vesicatoria*) and root-knot nematodes (*Meloidogyne* spp.) further exacerbate crop losses (Keawmanee et al., 2025). The frequent use of chemical pesticides has led to resistance development in pathogens and a resurgence of certain pests, thereby complicating disease management.

Taken together, these constraints not only lower the productivity and profitability of chilli but also threaten its sustainability as a key spice crop. Addressing them requires integrated nutrient management, soil health restoration strategies and eco-friendly disease management approaches such as resistant cultivars, biocontrol agents and biofertilizers (Fenibo and Matambo, 2025).

## 4 Biofertilizers

Biofertilizers represent a sustainable and eco-friendly alternative to chemical fertilizers, playing a pivotal role in enhancing soil fertility, nutrient cycling and crop productivity. They are defined as formulation of living or dormant cells of effective microbe strains that, when applied to seeds, plant surface and soils, stimulate development by making vital nutrients more readily available to plants. Unlike chemical fertilizers, which supply nutrients directly, biofertilizers improve plant nutrition indirectly by mobilizing, fixing and solubilizing nutrients in the rhizosphere, thereby fostering a self-sustaining soil ecosystem (Shahzad et al., 2025).

The most widely recognized groups of biofertilizers include nitrogen fixers (e.g., *Rhizobium*, *Azotobacter*, *Azospirillum*, Cyanobacteria), phosphorus solubilizers (e.g., *Bacillus*, *Pseudomonas*, *Penicillium*), potassium mobilizers (e.g., *Frateuria aurantia*) and PGPF such as *Trichoderma* spp., *Penicillium* spp., *Talaromyces* spp., *Phoma* and AMF (Figiel et al., 2025). These microorganisms contribute significantly to plant nutrition: nitrogen fixers convert atmospheric nitrogen into bioavailable ammonium, phosphate solubilizers release bound forms of phosphorus through organic acid production, while AMF enhance nutrient and water uptake through extensive hyphal networks (Kumar et al., 2022).

The significance of biofertilizers extends beyond nutrient enrichment. Their application contributes to soil health restoration by improving microbial diversity, organic matter decomposition and nutrient recycling. In degraded or intensively cultivated soils where chemical fertilizer use has led to nutrient imbalance and reduced microbial activity, biofertilizers act as key agents in restoring ecological balance (Samantaray et al., 2024). Furthermore, biofertilizers are cost-effective, reduce dependency on synthetic inputs and minimize environmental concerns such as nitrate leaching, eutrophication and greenhouse gas emissions, aligning with the global drive toward sustainable agriculture (Jasiński et al., 2026). Despite their proven potential, large-scale adoption of biofertilizers faces several challenges. These include variability in field performance, lack of region-specific formulations, limited shelf-life of microbial inoculants and insufficient farmer awareness. Recent advances in microbial genomics, nanotechnology and carrier material innovations however, are addressing these limitations by enabling the development of multi-strain consortia, bio-encapsulation techniques and stress-tolerant microbial strains that ensure higher efficacy and consistency under field conditions (Kanishka et al., 2025).

In the context of chilli (*C. annuum*), biofertilizers hold particular promise, given the crop’s high nutrient demand and sensitivity to soil health deterioration. Integration of PGPR and PGPF in chilli production systems not only improves nutrient uptake but also enhances tolerance to phytopathogens. This makes biofertilizers a critical component in sustainable chilli cultivation strategies (Rani et al., 2025).

### 4.1 Plant growth-promoting microorganisms

Plant Growth-Promoting Microorganisms (PGPM) encompass a diverse group of beneficial bacteria and fungi that inhabit the rhizosphere or endophytic compartments of plants and exert a positive influence on growth and productivity (Hakim et al., 2021). They are considered the functional core of biofertilizers because of their ability to enhance nutrient acquisition, modulate plant physiology and improve tolerance to both biotic and abiotic stresses (Chaudhary et al., 2022). PGPMs are broadly classified into two main categories: PGPR and PGPF. PGPR, such as *Pseudomonas*, *Bacillus*, *Azospirillum* and *Burkholderia* spp., colonize the rhizosphere and establish beneficial interactions with host roots, whereas PGPF, including AMF, *Trichoderma*, and *Piriformospora indica*, form either symbiotic or associative relationships that support nutrient uptake and pathogen resistance (Ansabayeva et al., 2025; Doddavarapu et al., 2024).

**Figure 1:**
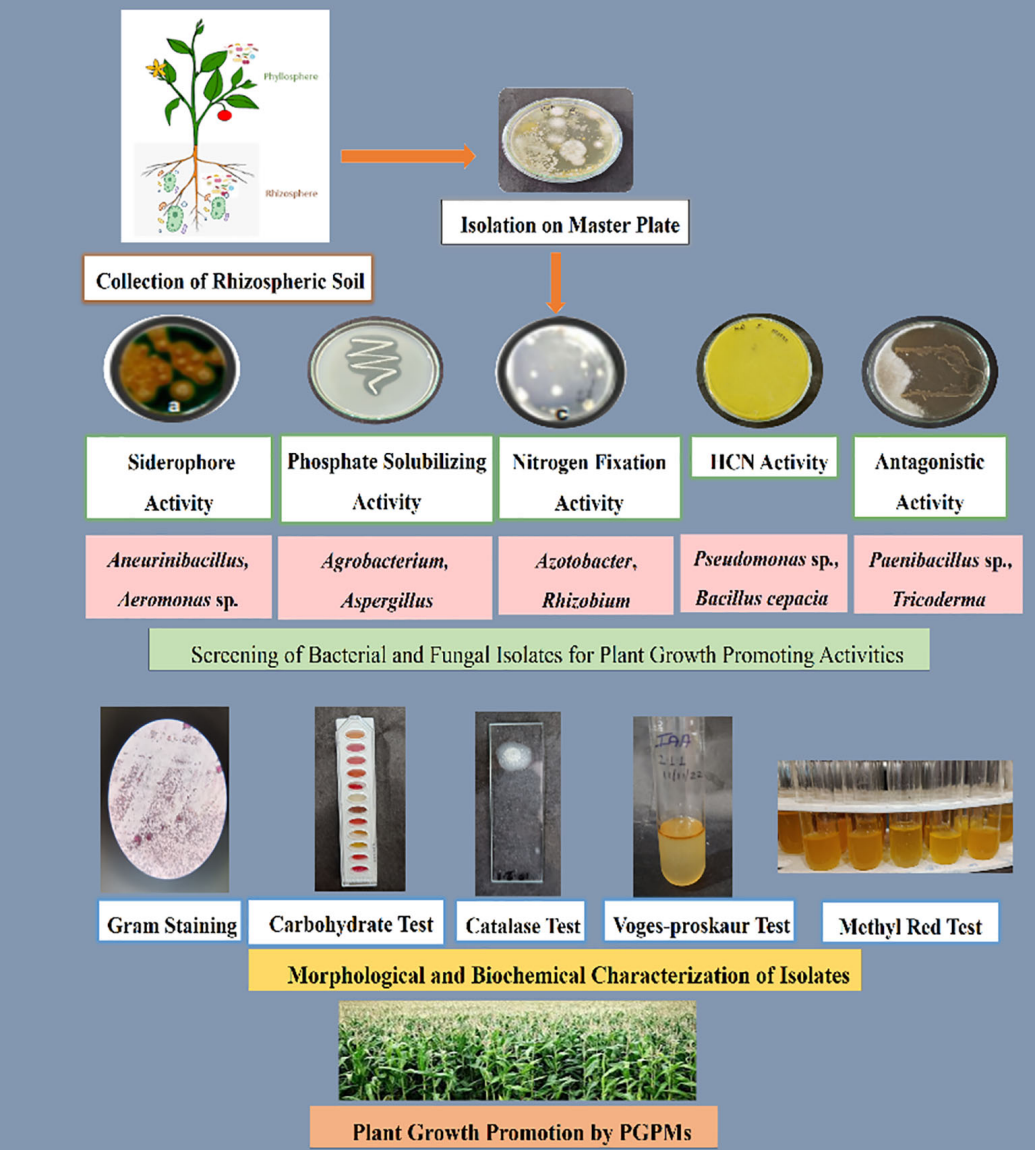
Schematic representation of the systematic isolation, functional screening, and comprehensive characterization of plant growth-promoting microorganisms (PGPMs) from rhizospheric soil.

The ecological role of PGPM is central to the maintenance of soil health and plant productivity. They contribute to nutrient cycling, organic matter decomposition and the stabilization of soil structure, thereby sustaining the functional diversity of agroecosystems (Laishram et al., 2025). Importantly, PGPMs act as keystone organisms that mediate plant soil-microbe interactions, ensuring resilience under conditions of nutrient deficiency, pathogen attack, abiotic stress such as drought and salinity (Pattnaik et al., 2021). Their multifunctionality positions them as an indispensable component in sustainable agricultural strategies, especially in crops like chilli, which demand high nutrient input and are susceptible to soilborne pathogens (Chen et al., 2024). The mechanisms through which PGPM promote plant growth can be broadly classified as direct and indirect. Direct mechanism primarily enhances nutrient availability and plant metabolism. These include biological nitrogen fixation, solubilization of insoluble phosphates and potassium, production of siderophores for iron acquisition, and secretion of phytohormones such as IAA, gibberellins, cytokinins and abscisic acid that regulate root and shoot development (**Figure 1**) (Dos Santos et al., 2020). Additionally, some PGPR possess ACC deaminase activity, which lowers ethylene levels in plants and thereby mitigates stress-induced growth inhibition (Orozco-Mosqueda et al., 2020).

Indirect mechanisms involve protection against pathogens and abiotic stressors. Many PGPR and PGPF act as natural biocontrol agents by producing antibiotics, lytic enzymes, volatile organic compounds and secondary metabolites that inhibit phytopathogens. *Trichoderma* spp. *Bacillus* and *Pseudomonas fluorescens* are known for their antagonistic activity against fungal diseases in chilli such as wilt, *Alternaria* Leaf Spot and Fruit Rot (Wang et al., 2024). Furthermore, PGPM can induce systemic resistance in plants by activating defense pathways involving salicylic acid, jasmonic acid and ethylene signaling. They also enhance tolerance to abiotic stresses by improving osmotic adjustment, producing stress-responsive metabolites and maintaining ion homeostasis (Arora and Fatima, 2022).

Collectively, PGPM represent an integrated solution to the double challenges of productivity enhancement and environmental sustainability (**Table 1**). Their classification into bacteria and fungi not only highlights their functional diversity but also underscores the need for their combined application as microbial consortia (Ansabayeva et al., 2025). For chilli cultivation, where nutrient deficiencies and soil-borne diseases often limit yield potential, PGPM offer a promising strategy by synergistically enhancing nutrient uptake, improving stress tolerance and suppressing pathogens. Thus, understanding their classification, ecological roles and underlying mechanisms forms a critical basis for developing next-generation biofertilizers tailored to this economically significant crop (Mahmud et al., 2021).

**Table 1:** Effect of biofertilizers (PGPR and PGPF) on growth parameters of different crops.

Biofertilizer	Crop	Properties	References
PLANT GROWTH PROMOTING RHIZOBACTERIA (PGPR)			
*Bacillus amyloliquefaciens, B. pumilus*,	*Solanum lycopersicum*	Improved plant growth, yield, and nutrient uptake.	(Bottini et al., 2004)
*Azospirillium lipoferum, Pseudomonas fluorescens* and *P. putida*	*Zea mays*	Increased root biomass, biomass and productivity.	(Noumavo et al., 2013)
*Bacillus* spp., *Providencia* spp. and *Brevundimonas diminuta, B. polymyxa, Azotobacter chroococcum* and *A. barasilense*	*Triticum aestivum*	Increased grain yield, raw protein and mineral nutrient concentration of wheat grains (P, K, Cu, Fe, Zn, Mn). Further, soil quality was also improved indicated by increased soil enzyme activities.	(Kumar Singh et al., 2022)
*Bacillus amyloliquefaciens, Bradyrhizobium japonicum*	*Glycine max*	Enhanced the ability to colonize plant roots and increase the number of nodules.	(Cheng et al., 2025)
*Mesorhizobium,A. chroococcum, P. aeruginosa*	*Cicer arietinum*	Enhanced growth, yield and phytopathogen growth inhibition.	(Naz et al., 2023)
*Burkholderia cepacia, B. amyloliquefaciens, Serratia marcescens, P. aeruginosa*	*Zingiber officinale*	Higher sprouting and lower disease incidence and greater rhizome yield were observed.	(Dinesh et al., 2015)
*Rhizobium bacteria, Azotobacter* spp.*, Azospirillum, Pseudomonas* spp., and *Azospirillum* spp.	*Vigna radiata*	Increased root and shoot length, germination percentage and vigor index of seeds.	(Mitra et al., 2021)
*P. aeruginosa, P. putida*, and *P. fluorescens*	*Oryza sativa*	Treatment combinations increased grain yield, plant growth, nutrient contents in grain and straw of rice.	(Nandakumar et al., 2001)
PLANT GROWTH PROMOTING FUNGI (PGPF)			
*T. Harzianum* and *Glomus intraradices*	*Solanum lycopersicum*	Improvement in yield and quality by increased total soluble solids, ascorbic acid, β-carotene, phosphorus, manganese content etc.	(Sani et al., 2020)
*T. asperellum*	*Cicer arietinum* and *Phaseolus vulgaris*	Enhanced seed germination and plant growth for both plants.	(Vijayakumar et al., 2025)
*T. harzianum* and *T. atroviride*	*Pisum sativum* and *Brassica napus*	Elevated Plant growth.	(Garstecka et al., 2023)
*Aspergillus awamori S19*	*Vigna radiata*	Phosphate solubilization.	(Ali et al., 2021)
*Pleurotus tuber-regium, Lentinus squarrosulus* and *Ganoderma* sp.	*Triticum aestivum* and *Solanum lycopersicum*	The growth parameters of both plants were increased by chitinase and esterase activities, siderophore production and phosphate solubilization by fungal strains.	(Mensah et al., 2018)

## 5 Enzymatic activities of plant growth-promoting microorganisms

The enzymatic diversity of PGPM is central to their ecological success in the rhizosphere and their role as biofertilizers (Kumar et al., 2025). These microbes secrete a variety of extracellular enzymes that accelerate the degradation of complex organic matter, thereby releasing nutrients in plant-available forms and simultaneously influencing rhizospheric interactions. Among these amylases, proteases, cellulases, lipases and ureases represent the most functionally significant groups (Wei et al., 2024).

Amylases contribute to carbon cycling by hydrolyzing starch and polysaccharides into simpler sugars, which not only enrich soil organic carbon pools but also provide readily assimilable substrates that stimulate microbial proliferation and root colonization (Sritongon et al., 2023). Proteases secreted by organisms such as *Bacillus, Trichoderma*, and *Aspergillus* hydrolyze proteins into peptides and amino acids, thereby enhancing nitrogen turnover. These enzymes also serve as defensive tools by degrading the proteinaceous structures of phytopathogens, thereby exerting a biocontrol effect in addition to nutrient release (Song et al., 2023). Similarly, cellulases, produced by PGPMs, catalyze the breakdown of cellulose-rich plant residues into glucose monomers. This process contributes significantly to organic matter recycling and soil fertility enhancement, while also facilitating microbial colonization of plant roots by modifying plant surface structures (Hussain et al., 2025).

Lipases play a relatively less explored but equally important role in soil systems. By breaking down complex lipids into fatty acids and glycerol, lipase-producing microorganisms accelerate the decomposition of lipid-containing residues and generate metabolites that serve as energy-rich substrates for microbial communities. Furthermore, lipases have been associated with antagonistic interactions, in which they disrupt the lipid membranes of phytopathogens, thereby enhancing plant protection (Wang et al., 2024). On the other hand, ureases, which are produced by bacteria like *Rhizobium*, *Klebsiella* and *Bacillus* are key drivers for the nitrogen cycle in soils. Urease catalyzes the hydrolysis of urea into ammonia, which is subsequently converted into plant-available nitrogen forms. This activity ensures more efficient utilization of applied urea fertilizers while reducing nitrogen losses through volatilization and leaching, thereby improving nutrient-use efficiency (Phang et al., 2018).

Collectively, these enzyme-mediated processes extend beyond nutrient mineralization to play a crucial role in shaping plant-microbe-soil interactions. The release of low-molecular-weight metabolites during enzymatic hydrolysis often functions as signaling molecules that stimulate root exudation and microbial recruitment, creating a feedback loop that strengthens rhizospheric cooperation (Tian et al., 2024). The cumulative effect of enzymatic activities is the establishment of a biologically active soil environment where nutrient cycling, plant growth and disease resistance are simultaneously enhanced. In crops like chilli, which are highly responsive to rhizospheric microbial dynamics, enzyme-mediated processes significantly contribute to yield stability under conditions of nutrient limitation, pathogen pressure and soil degradation (Das and Varma, 2010).

Thus, the enzymatic activities of PGPM represent a critical mechanism underpinning their biofertilizer potential. By coupling organic matter degradation with nutrient mobilization, root colonization and pathogen suppression, these microorganisms serve as natural soil engineers that enhance plant productivity in a sustainable and environmentally compatible manner (Ademola et al., 2024).

## 6 Mechanisms of plant growth promotion by biofertilizers

The effectiveness of biofertilizers lies in their multifaceted mechanisms that enhance plant growth both directly and indirectly. Understanding these mechanisms is critical to appreciating their potential as sustainable alternatives to chemical inputs (Chaudhary et al., 2022).

A fundamental process underlying biofertilizer action is biological nitrogen fixation, carried out by diazotrophic microorganisms such as *Rhizobium*, *Azotobacter* and *Azospirillum*. These microbes convert atmospheric nitrogen (N ₂) into ammonium (NH_4_^+^), a plant-assimilable form, via the nitrogenase enzyme complex (Kumar et al., 2022). This process reduces dependency on synthetic nitrogen fertilizers and ensures a continuous supply of nitrogen in the rhizosphere. Complementing nitrogen fixation is phosphorus solubilization, mediated by phosphate-solubilizing bacteria (PSB) such as *Bacillus* and *Pseudomonas* (Silva et al., 2023). These microorganisms secrete organic acids (gluconic, citric and oxalic) and phosphatases that mobilize insoluble phosphates, thereby enhancing phosphorus availability to plants (Bhattacharjya et al., 2021). Similarly, potassium solubilization is achieved by microbes like *Frateuria aurantia* and certain fungal taxa, which release organic acids and chelating compounds that transform potassium bearing minerals into plant-accessible forms, significantly contributing to improved plant vigor and stress tolerance (Rajawat et al., 2019).

Another crucial mechanism is siderophore production, which enhances iron acquisition in iron-limited soils. Siderophores are low molecular-weight, high affinity iron-chelating compounds secreted by bacteria such as *Pseudomonas* and *Burkholderia* (Albelda-Berenguer et al., 2019). By binding Fe³ ⁺ and transporting it back to microbial or plant cells, siderophores not only supply iron but also competitively exclude pathogenic microorganisms from accessing this essential micronutrient, thereby exerting a dual role in nutrition and biocontrol (Xie et al., 2024). In addition to nutrient mobilization, PGPM play a central role in phytohormone production. Auxins such as IAA, gibberellins (GA) and cytokinins synthesized by microbe’s influence root architecture, stimulate cell elongation and regulate shoot growth. IAA production promotes root proliferation, lateral root emergence and root hair development, enabling greater soil exploration and nutrient uptake (Andrade et al., 2023).

PGPM also enhance soil nitrogen dynamics by releasing ammonium through mineralization processes, while some species produce hydrogen cyanide (HCN) as a secondary metabolite. HCN production in the rhizosphere acts as a biocontrol trait by inhibiting pathogenic fungi, despite being toxic in higher concentrations (Sehrawat et al., 2022). Micronutrient mobilization is further advanced through zinc solubilization, wherein microorganisms convert insoluble forms such as zinc oxide and zinc carbonate into soluble Zn² ⁺ via the action of organic acids and chelators, thereby addressing widespread zinc deficiencies in agricultural soils (Yadav et al., 2020). The antagonistic potential of biofertilizers is further strengthened through the production of antibiotics (e.g., Phenazines, Pyrrolnitrin and Iturins), which inhibit phytopathogens by interfering with their cell wall integrity and metabolic pathways (Sindhu et al., 2022). Likewise, PGPM synthesize lytic and protective enzymes, including chitinases, glucanases, cellulases and proteases, that degrade the structural components of pathogenic fungi and bacteria. These enzymes not only suppress diseases but also promote root colonization and symbiotic establishment (Das and Mukherjee, 2021). In addition, many rhizosphere microorganisms produce EPSs, which form biofilms around roots. EPSs improve soil aggregation, enhance water retention and protect plants against desiccation, salinity and heavy metal stress, while also serving as signaling molecules in plant-microbe communication (Kumar et al., 2025).

**Figure 2:**
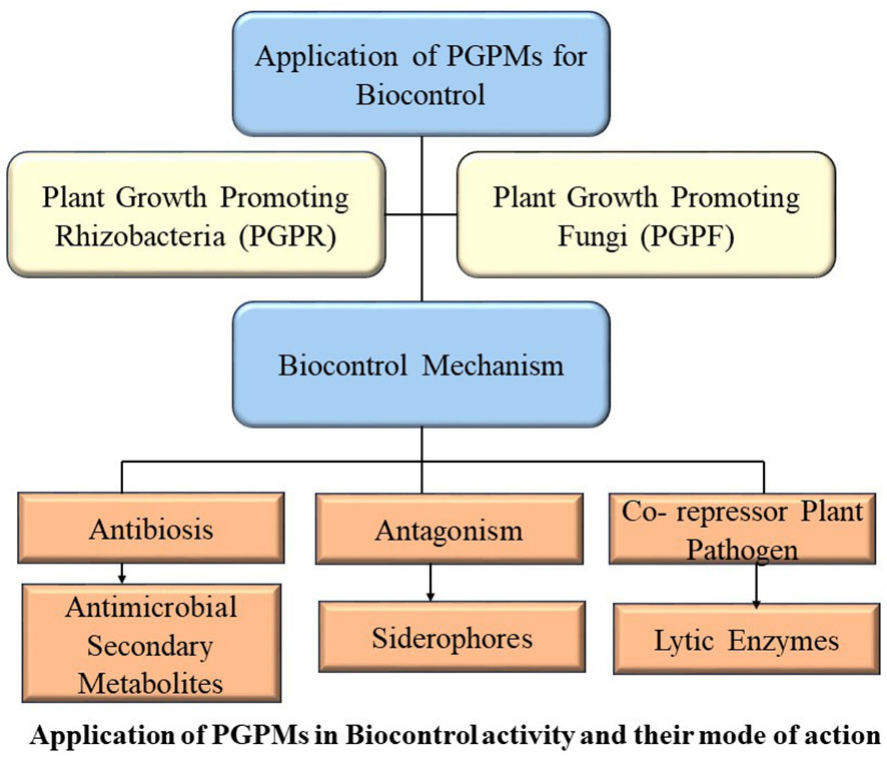
Integrated conceptual framework delineating the mechanistic roles of plant growth-promoting microorganisms (PGPMs) in the biological control of phytopathogens.

To withstand abiotic stresses, biofertilizers synthesize osmoprotectants, such as proline, glycine betaine and trehalose, which stabilize proteins and membranes under drought, salinity and heat stress. These compounds not only safeguard microbial cells but also indirectly enhance plant tolerance to adverse environments (Rani et al., 2024). Furthermore, the microbial induction of antioxidant metabolism plays a pivotal role in mitigating oxidative stress. PGPM helps plants neutralize reactive oxygen species (ROS) produced during environmental challenges by promoting plant antioxidant enzymes like superoxide dismutase (SOD), catalase (CAT), and peroxidases (POD). This preserves cellular homeostasis and maintains metabolic activity (Rao et al., 2025). Taken together, these mechanisms illustrate the multifactorial nature of biofertilizer-mediated plant growth promotion. Rather than acting through a single pathway, PGPM employ a synergistic combination of nutrient mobilization, hormone modulation, pathogen suppression, stress alleviation and soil structure enhancement (**Figure 2**) (Kumar et al., 2022). This integrated mode of action not only boosts crop productivity but also contributes to long-term soil health and resilience, making biofertilizers an indispensable component of sustainable agriculture (**Table 2**) (Kabato et al., 2025).

**Table 2:** Comparative evaluation of single and co-inoculation of PGPR and PGPF on growth, yield, nutrient uptake, and disease suppression in chilli (*C. annuum*).

Microbial treatment	Type of inoculation	Major observations in chilli (*C. annuum*)	Comparative outcome	References
*Pseudomonas* sp.	Single inoculation	Improved plant growth and yield parameters under pot culture conditions	Moderate increase in yield compared to control	(Pandey and Gupta, 2020)
*Bacillus* sp.	Single inoculation	Enhanced vegetative growth and nutrient utilization	Lower performance than consortium treatment	(Adjei et al., 2025)
*Pseudomonas* sp. + *Bacillus* sp.	Co-inoculation	Highest green chilli yield (26.75 t ha^-^¹) with improved plant vigor and nutrient uptake	Outperformed individual inoculations and recommended fertilizer treatment	(Narayanan and Madhavan, 2020)
*Piriformospora indica*	Single inoculation	Enhanced root colonization and plant growth	Growth promotion observed but lower than combined inoculation	(Anjana et al., 2023)
*Pseudomonas fluorescens*	Single inoculation	Increased vegetative growth and rhizosphere activity	Moderate growth enhancement	(Jiménez et al., 2020)
*Piriformospora indica* + *Pseudomonas fluorescens*	Co-inoculation	Significant enhancement in plant height, branching, root colonization, and overall growth performance	Synergistic interaction produced superior results compared with single inoculations	(Meena et al., 2010)
Indigenous PGPF isolates (NBP-61 and others)	Single inoculation	Enhanced seed germination, plant growth, and anthracnose resistance	Disease protection up to 78.75%	(Naziya et al., 2019)
*Bacillus subtilis* + *Bacillus pumilus*	Co-inoculation	Increased transplant vigor, survival, disease resistance, and fruit yield in chilli system	Fruit yield increased substantially with reduced damping-off and anthracnose incidence	(Kaushal et al., 2019)
AM fungus (*Acaulospora* sp.)	Single inoculation	Improved nutrient uptake and root growth	Beneficial but less effective than combined application	(Begum et al., 2019)
AM fungus + PGPR consortium	Co-inoculation	Improved growth, biomass accumulation, and nutrient acquisition	Combined inoculation showed synergistic improvement over single inoculation	(Barzegari Barogh et al., 2023)
*Azospirillum brasilense*	Single inoculation	Enhanced root development and chlorophyll content	Improved seedling vigor and nutrient absorption	(Zeng et al., 2024)
*Trichoderma harzianum*	Single inoculation	Improved seed germination and suppression of soil-borne pathogens	Enhanced disease resistance and moderate yield increase	(Asghar et al., 2024)
*Azotobacter chroococcum*	Single inoculation	Increased shoot length, root biomass, and nutrient uptake	Improved vegetative growth compared with uninoculated control	(Van Oosten et al., 2018)
*Pseudomonas fluorescens* + *Trichoderma harzianum*	Co-inoculation	Enhanced plant growth, induced systemic resistance, and reduced fungal disease incidence	Consortium treatment showed stronger biocontrol efficacy and yield improvement	(Sandheep et al., 2013)
*Bacillus subtilis* + AM fungi	Co-inoculation	Increased phosphorus uptake, biomass accumulation, and fruit yield	Synergistic interaction improved nutrient-use efficiency over single inoculations	(Severo et al., 2025)
*Azospirillum brasilense* + *Pseudomonas fluorescens*	Co-inoculation	Improved root architecture, flowering, and fruit production	Higher yield and nutrient uptake than individual microbial treatments	(Pérez-Rodríguez et al., 2020)
*Trichoderma asperellum* + *Bacillus velezensis*	Co-inoculation	Enhanced antioxidant activity, growth promotion, and disease suppression	Superior plant performance under biotic stress conditions	(Zhou et al., 2021)
PGPR consortium (*Bacillus*, *Pseudomonas*, and *Azospirillum* spp.)	Co-inoculation	Improved soil microbial activity, nutrient mobilization, and chilli productivity	Consortium exhibited maximum synergistic effect on growth and yield	(Terra et al., 2024)

The table summarizes the synergistic effects of microbial consortia compared with individual inoculations, highlighting their potential application in sustainable chilli cultivation.

## 7 Preparation of bioformulation using selected fungal and bacterial isolates

The successful application of PGPM, including both fungal and bacterial isolates, largely depends on their formulation into stable and viable bioinoculants (Monica et al., 2025). A bioformulation is essentially a delivery system that ensures prolonged survival, easy handling and effective field performance of microbial inoculants. Unlike pure culture suspensions, which are highly perishable and sensitive to environmental fluctuations, bioformulations utilize carriers and additives that not only support the persistence of microbes but also improve their compatibility with agricultural practices (Khan et al., 2023).

### 7.1 Carriers and additives

Carriers serve as protective substrates that harbor the microbial inoculum, maintain adequate moisture and enhance the inoculant’s delivery to the plant rhizosphere. An ideal carrier material should be sterile, non-toxic, readily available, cost-effective and capable of supporting the microbial population over extended storage (Sivaram et al., 2023). Among commonly used carriers, talc is favored for its inert nature, fine texture and good adhesion properties, enabling uniform seed coating. Lignite, with its high organic matter and porosity offers an excellent environment for microbial survival by retaining moisture and nutrients (Afzal et al., 2020). Charcoal has also been widely used for its strong adsorptive capacity, which stabilizes metabolites secreted by microbes and protects inoculants from desiccation. Additionally, compost-based carriers provide not only a habitat but also supplemental nutrients, creating a synergistic effect when applied to the soil (Makoto and Koike, 2021). Often, additives such as gum Arabic, polyvinylpyrrolidone (PVP), or sugars are incorporated into formulations to improve adhesion, enhance microbial viability and facilitate seed or soil application (Monisha et al., 2023).

### 7.2 Shelf life and survival of microbial inoculants

The long-term efficacy of bioformulations is determined by the shelf life of microbial inoculants, which reflects their ability to remain viable and metabolically active under storage and field conditions. Shelf life is influenced by the type of microorganism, formulation method and carrier properties (Priya et al., 2025). Bacterial inoculants such as *Rhizobium*, *Pseudomonas* and *Bacillus* typically require carriers that buffer against desiccation and temperature fluctuations, while fungal inoculants like *Trichoderma* and mycorrhizal fungi demand carriers with high aeration and organic matter to sustain spore viability. Moisture retention, pH stability and protection from ultraviolet (UV) radiation are critical factors that dictate the survival dynamics of the inoculated strains (Sivaram et al., 2023).

Research has demonstrated that talc and charcoal-based formulations can sustain microbial populations for 6–12 months, depending on storage conditions, whereas compost-based formulations tend to have shorter shelf lives but higher field efficacy due to synergistic interactions with native soil microbiota (Kumar et al., 2013). Advances in encapsulation techniques, such as alginate bead entrapment and polymer-coated formulations, are being increasingly explored to enhance the survival, controlled release and shelf stability of inoculants (Riseh et al., 2021). Such innovations in bioformulation technology ensure that the selected bacterial and fungal isolates not only reach the target crop but also remain physiologically active to exert their plant growth-promoting and biocontrol effects (Khan et al., 2023).

## 8 *In vivo* pot experiment to evaluate bacterial-fungal biofertilizer combinations and identify the best-performing inoculant for chilli

The shift toward sustainable nutrient management in intensive horticulture needs biologically efficient alternatives to synthetic inputs (Azharuddin Scholar et al., 2025). Plant growth-promoting microorganisms (PGPM), enhance plant productivity via nutrient solubilization, phytohormone production, nitrogen fixation, and induced systemic resistance (Ansabayeva et al., 2025). However, the consistency of these effects under natural conditions remains limited, as *in vitro* assays fail to capture the complexity of rhizosphere interactions. Consequently, *in vivo* pot experiments are essential for validating the functional efficacy and ecological competence of microbial inoculants prior to field application (Díaz-Rodríguez et al., 2025). *In vivo* assays conducted under controlled pot conditions enable the systematic evaluation of microbial inoculants with respect to plant growth promotion, nutrient acquisition efficiency, and rhizosphere colonization dynamics (Shahwar et al., 2023). Such experimental systems facilitate the quantification of key agronomic parameters, including germination index, shoot and root biomass accumulation, chlorophyll content, and nutrient uptake profiles, thereby providing a comprehensive assessment of inoculant performance (Calvo et al., 2017). Furthermore, these studies are particularly relevant for investigating the compatibility and synergistic interactions between bacterial and fungal partners in consortium-based formulations. The integration of functionally complementary microorganisms has been shown to enhance biofertilizer efficacy through mechanisms such as cooperative nutrient mobilization, niche differentiation, and improved rhizosphere persistence (Singh et al., 2025).

The formulation strategy plays a decisive role in determining the viability, shelf life, and field performance of microbial inoculants. Among various carrier materials, talc-based formulations have gained prominence due to their physicochemical stability, low cost, and ability to support high microbial cell densities (Gopalakrishnan et al., 2016). Talc powder acts as an inert carrier matrix, providing a conducive microhabitat for microbial survival during storage and facilitating efficient delivery to the rhizosphere (Gopalakrishnan et al., 2016). The incorporation of carboxymethyl cellulose (CMC) as a binding agent enhances the adhesion of microbial cells to seed surfaces, thereby improving inoculation efficiency and initial colonization. Additionally, calcium carbonate (CaCO ₃) is commonly employed as a buffering agent to stabilize the pH of the formulation, thereby maintaining microbial viability and metabolic activity over prolonged storage periods (Ramlucken et al., 2020).

The combined application of bacterial and fungal inoculants is increasingly recognized as a robust strategy for improving crop performance, particularly in nutrient-limited soils (Al Methyeb et al., 2023). Bacterial strains, including phosphate-solubilizing and PGPR, contribute to the mobilization of essential nutrients and synthesis of growth regulators, whereas fungal partners, such as phosphate-solubilizing fungi and mycorrhiza-like organisms, enhance nutrient uptake efficiency and root system architecture (Hasan et al., 2024). This functional complementarity is especially pertinent for chilli (*C. annuum*), a high-value solanaceous crop characterized by substantial nutrient demand and sensitivity to soil fertility constraints (Otieno et al., 2021).

Particularly, regarding to this framework, there was a controlled *in vivo* pot experiment reported to evaluate the efficacy of selected bacterial-fungal consortia formulated using a talc-based carrier system supplemented with CMC and CaCO ₃. The experimental design allows the comparison of different inoculant combinations in terms of plant growth promotion and physiological performance for the characterization of the most effective biofertilizer consortium for chilli cultivation.

## 9 Role of plant growth-promoting microorganisms in growth enhancement of chilli

**Figure 3:**
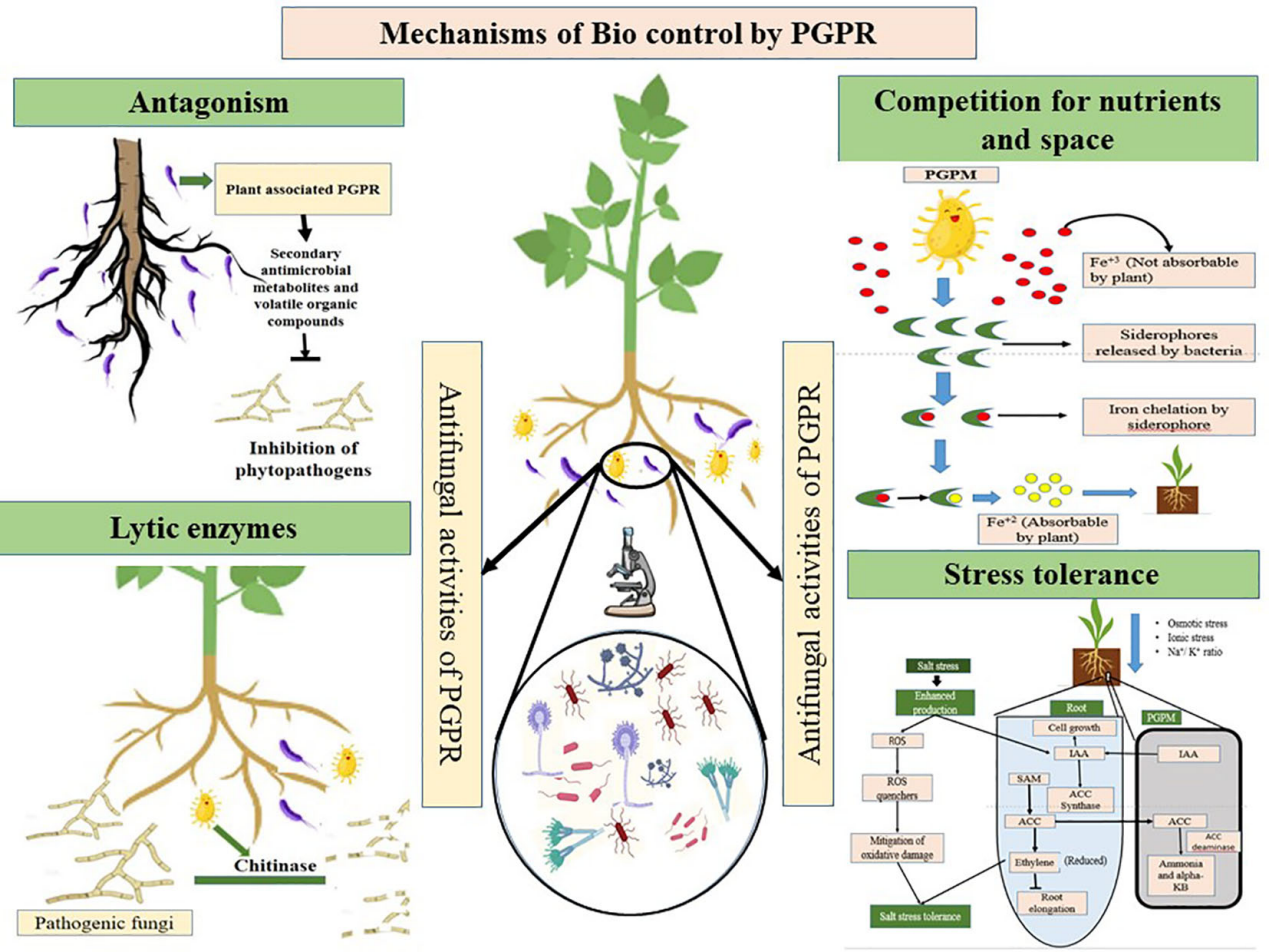
Mechanistic framework of biocontrol and plant growth-promoting activities mediated by plant growth-promoting microorganisms in the rhizosphere.

In chilli (*C. annuum* L.), a crop of high nutritional, medicinal and economic significance which productivity is often constrained by poor nutrient-use efficiency, recurrent soil-borne pathogens and environmental stresses such as salinity, drought and temperature fluctuations. PGPM offer a promising eco-biological alternative to overcome these limitations by enhancing growth through multifaceted mechanisms that operate at physiological, biochemical and ecological levels (Yaish and Halo, 2026). One of the primary contributions of PGPM in chilli lies in their ability to optimize root system architecture. Rhizobacteria such as *Azospirillum* and *Pseudomonas* release IAA and volatile organic compounds (VOCs), which stimulate root branching and elongation (Hyder et al., 2020). Enhanced root proliferation in turn improves nutrient foraging efficiency, particularly for nitrogen and phosphorus, which are critical for vegetative growth and fruit set in chilli. Similarly, mycorrhizal fungi extend the absorptive surface area through extraradical hyphae, facilitating uptake of phosphorus, potassium and micronutrients that directly influence capsaicinoid biosynthesis and fruit quality (Wahab et al., 2023). PGPM also exert effects on the reproductive physiology of chilli. Reports have demonstrated that inoculation with *Trichoderma* spp. and *Bacillus* spp. not only improves plant vigor but also significantly increases fruit number and size, contributing to higher marketable yield. Another key dimension is the resilience conferred by PGPM under stress-prone cultivation environments (Chouhan et al., 2021). Extracellular polysaccharides and osmoprotectants secreted by beneficial microbes create a buffered rhizospheric environment that improves soil aggregation, water retention and tolerance to osmotic stress (Das et al., 2025). For chilli grown in semi-arid and marginal lands, this capacity translates into better survival, sustained flowering and higher yield stability. Additionally, PGPM trigger antioxidant defense systems in chilli by upregulating enzymes like superoxide dismutase, peroxidase and catalase, which protect reproductive tissues from oxidative damage during heat or drought (Khaskheli et al., 2025). Equally important is their role in disease suppression, which indirectly enhances chilli growth. Pathogens such as *Alternaria alternata, Fusarium oxysporum*, *Phytophthora capsici* and *Colletotrichum* spp. frequently devastate chilli fields, leading to severe yield losses (Delai et al., 2024). PGPM produce antibiotics, siderophores and lytic enzymes that inhibit pathogen colonization by reducing the metabolic burden of plant defense and PGPM allow chilli plants to allocate greater energy toward growth and fruiting (**Figure 3**) (David et al., 2018).

The growth-promoting effects establish PGPM as crucial allies in chilli cultivation. They do not merely act as nutrient providers but function as regulators of plant developmental program, enhancer of stress resilience and defender against pathogenic challenge (Ansari et al., 2024). Their role in chilli growth enhancement exemplifies the transition from input-intensive agriculture toward biologically integrated sustainable system, where microbial partnership unlocks the latent yield potential and improves both quality and productivity of this globally important spice crop (Siddiki et al., 2025).

## 10 Conclusion

The growing need for sustainable intensification of chilli (*C. annuum*) production highlights the importance of biologically driven strategies that improve nutrient-use efficiency, soil functionality, and crop resilience. In this context, PGPM, particularly PGPR and PGPF, represent promising components of advanced biofertilizer systems due to their roles in nutrient mobilization, phytohormone production, rhizosphere modulation, stress alleviation, and pathogen suppression. Increasing evidence indicates that consortium-based bacterial-fungal inoculants often outperform single-strain formulations by enhancing rhizosphere competence, nutrient acquisition, root development and overall plant performance. However, large-scale applications remain limited by challenges including poor rhizosphere colonization, reduced inoculant viability, carrier incompatibility, and inconsistent performance under diverse environmental conditions. Future research should focus on developing stable multi-strain formulations with optimized carriers, improved shelf life, ecological adaptability, and functional compatibility under field conditions. Integrating advances in microbial ecology, formulation technology, and host-microbe interaction studies will be essential for improving the agronomic reliability of PGPR-PGPF consortia for sustainable chilli production and long-term soil health improvement.
